# Opium Intoxication

**Published:** 1875-07

**Authors:** 


Opium Intoxication.—Dr. Joseph Parrish says:

To relieve the symptoms it is desirable to avoid the shock, as it is desirable to avoid it in surgical operations.

For this purpose the practitioner should immediately reduce the accustomed supply to the minimum dose which will meet this condition.

When the minimum is reached, the suffering of the patient begins, and then the practice should be to give tone to the nervous system, as the opium stimulus is withdrawn. The reduction should be in minute quantities, and the tonic doses full and persistent.

The moral sentiment, the confidence and courage of the patient, should at all times be kept up to the attainable degree.

